# Supplementary material for: Copy number variation of two begomovirus acquired and inoculated by different cryptic species of whitefly, Bemisia tabaci in Okra
Source: PLoS One. 2022 Mar 30;17(3):e0265991. doi: 10.1371/journal.pone.0265991 (PMC8966996; doi:10.1371/journal.pone.0265991)
Supplement: S1 Fig — (DOCX) [file pone.0265991.s001.docx]

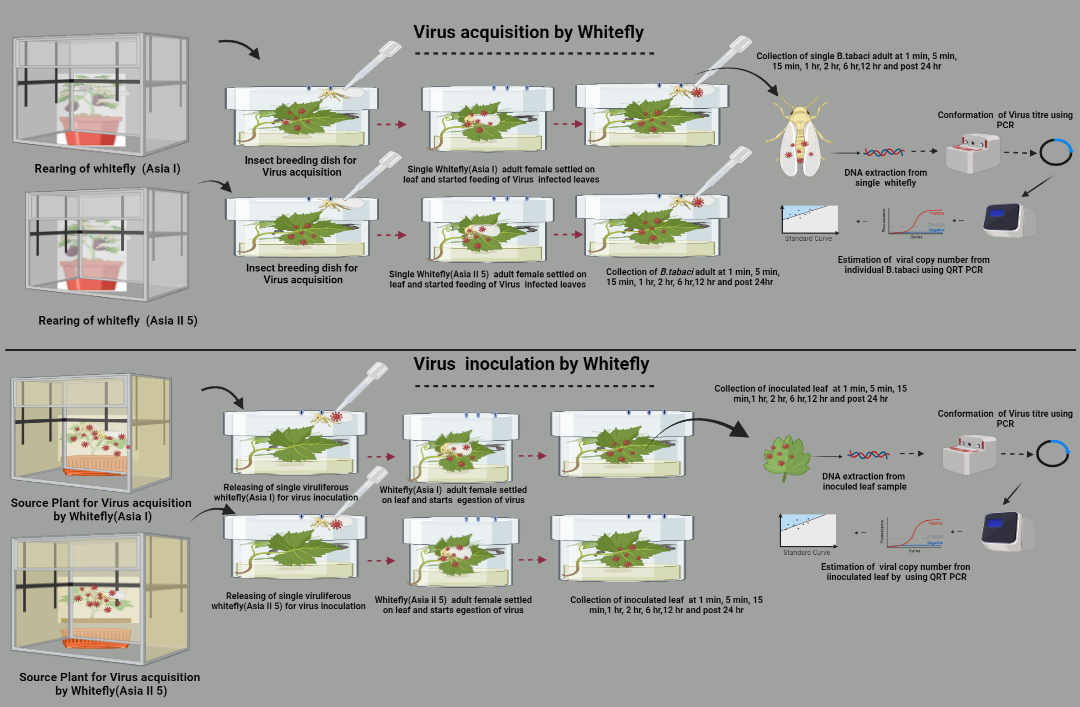


**S1 Fig.** Ingestion and egestion process of YVMV and OELCuV by single *B.tabaci* (both Asia I and Asia II 5) using detached leaf assay.
