# Supplementary material for: Copy number variation of two begomovirus acquired and inoculated by different cryptic species of whitefly, Bemisia tabaci in Okra
Source: PLoS One. 2022 Mar 30;17(3):e0265991. doi: 10.1371/journal.pone.0265991 (PMC8966996; doi:10.1371/journal.pone.0265991)
Supplement: Attachment — Submitted filename: 22.docx [file pone.0265991.s002.docx]

This study reports the variations in begomoviral acquisition and transmission by Asia I and Asia II 5 species of whiteflies. Real time PCR method is used at periodic intervals to quantify viral loads during ingestion and egestion by whiteflies. Significant variations are observed in virus uptake and transmission by both species for yellow vein mosaic & okra leaf curl virus. These variations indicate Asia I is better transmitter for OELCuV and Asia II 5 for YUMV which can significantly affect the virus pandemics. Overall, the manuscript is clearly written, data is clearly represented and results are explained well. However, I have few major questions which should be addressed before consideration for submission.

1- The authors did not mention about the sample size for example the number of whiteflies used for ingestion assay as well as for egestion assay. Please include these details for sample size for each timepoint in methods section.
2- There is no information about the sex ratio of the number whiteflies used for ingestion and egestion assay. As there are reports stating females feed and inoculate more over male whiteflies therefore considered better transmitters over males. If male to female ratios is not kept consistent then it can significantly affect the observations. Including this relevant information will add more vigor to the present observations.
3- Did authors check the expression of any endogenous genes to normalize basal levels?
4- Please explain term inoculation period which is used throughout the manuscript.
5- The authors have focused on acquisition and egestion of the virus but did not discuss about retention by whiteflies. Was there any data or observation made about retention of the virus?
6- Since there is significant amount of variation observed in virus uptake. Did authors perform any quantification by real time PCR to normalize the initial amounts of viruses used for feeding?
7- The sequences for the accession numbers OL743532 & OL743533 are not found in NCBI GenBank. Please update.
8- In figure 4 D data point for 15 min has large deviation than others. Please explain.
9- Did authors tried any other method of virus detection like western blot to add strength to this real time data?
