# Supplementary material for: Copy number variation of two begomovirus acquired and inoculated by different cryptic species of whitefly, Bemisia tabaci in Okra
Source: PLoS One. 2022 Mar 30;17(3):e0265991. doi: 10.1371/journal.pone.0265991 (PMC8966996; doi:10.1371/journal.pone.0265991)
Supplement: Attachment — Submitted filename: Review comments for Copy number variation of two begomovirus.docx [file pone.0265991.s003.docx]

The research article on “Copy number variation of two begomovirus acquired and inoculated by different cryptic species of whitefly, Bemisia tabaci in Okra" focuses mainly on the comparative study of 17 begomovirus species viz. yellow vein mosaic virus (YVMV) and okra enation leaf curl virus 18 (OELCuV) ingested and egested by two cryptic species (Asia I and Asia II 5) of B.tabaci at 19 different time interval using detached leaf assay. As such, the article might be of interest to a broad audience who might want an introduction to the management on crop improvement. I have enjoyed reading the entire manuscript except at a few places with typo errors and some sentences are very long and not appropriate. I advise authors to read again and correct them wherever necessary.
